# Correlates of thymus size and changes during treatment of children with severe acute malnutrition: a cohort study

**DOI:** 10.1186/s12887-017-0821-0

**Published:** 2017-03-14

**Authors:** Maren Johanne Heilskov Rytter, Hanifa Namusoke, Christian Ritz, Kim F. Michaelsen, André Briend, Henrik Friis, Dorthe Jeppesen

**Affiliations:** 1 Department of Nutrition, Exercise and Sports, University of Copenhagen, Rolighedsvej 30, 1958 Frederiksberg C, Denmark; 2 Mwanamugimu Nutrition Unit, Mulago Hospital, Kampala, Uganda; 3 Tampere Centre for Child Health Research, University of Tampere, Tampere, Finland; 4 Department of Pediatrics, Copenhagen University Hospital Hvidovre, Copenhagen, Denmark

**Keywords:** Re-feeding, Immune function, Undernutrition, Thymus, Electrolytes, Inflammation, Phosphate

## Abstract

**Background:**

The impairment of immune functions associated with malnutrition may be one reason for the high mortality in children with severe acute malnutrition (SAM), and thymus atrophy has been proposed as a marker of this immunodeficiency. The aim of this study was to identify nutritional and clinical correlates of thymus size in children with SAM, and predictors of change in thymus size with nutritional rehabilitation.

**Methods:**

In an observational study among children aged 6–59 months admitted with SAM in Uganda, we measured thymus area by ultrasound on hospital admission to treatment with F75 and F100, on hospital discharge and after 8 weeks of nutritional rehabilitation with ready-to-use therapeutic food, as well as in well-nourished healthy children. We investigated anthropometric, clinical, biochemical and treatment-related correlates of area and growth of the thymus.

**Results:**

Eighty-five children with SAM with a median age of 16.5 months were included. On admission 27% of the children had a thymus undetectable by ultrasound. Median thymus area was 1.3 cm^2^ in malnourished children, and 3.5 cm^2^ in healthy children (*p* < 0.001). Most anthropometric z-scores, hemoglobin and plasma phosphate correlated positively with thymus area. Thymus area correlated negatively with caretaker-reported severity of illness, plasma α-1 acid glycoprotein, and C-reactive protein >5 mg/L. At follow-up after 8 weeks, median thymus area had increased to 2.5 cm^2^ (*p* < 0.001). Increase in thymus area during treatment was associated with simultaneous increase in mid-upper-arm circumference, with 0.29 cm^2^ higher increase in thymus area per cm larger increment in MUAC (*p* = 0.03). Children whose F-75 had partially been replaced by rice porridge during their hospital admission had less increase in thymus area after 8 weeks.

**Conclusion:**

Malnutrition and inflammation are associated with thymus atrophy, and thymus area seems positively associated with plasma phosphate. Substituting therapeutic formula with unfortified rice porridge with the aim of alleviating diarrhea may impair regain of thymus size with nutritional rehabilitation. This calls for research into possible effects of phosphate status on thymus size and other immunological markers.

**Trial registration:**

The study is based on data from the FeedSAM study, ISRCTN55092738.

## Background

Severe acute malnutrition (SAM) in children is a life-threatening condition [1], and many hospitals in sub-Saharan Africa report mortality rates above 20% in children admitted with SAM [2]. The reasons for the high mortality are not clear, but most deaths are attributable to infectious diseases, probably facilitated by impaired immune function in the malnourished children [3]. Although the mechanism behind the immune deficiency of malnutrition is still poorly understood, one immunological alteration consistently reported in malnourished children is atrophy of the thymus [4–6]. As such, the size of the thymus has been suggested to be a marker of the immunodeficiency of malnutrition [3]. Even in absence of severe acute malnutrition, thymus size is associated with nutritional status, and independently of nutritional status, children with a small thymus have higher mortality [7, 8]. Although it is unknown to which extend thymus size reflects immune competence, the observations suggest that thymus size may be a marker of robustness in small children. This could potentially make it highly relevant to study in children with SAM, who are extremely vulnerable in the first place.

Even though malnourished children are known to have thymus atrophy, it is unknown how this is modified by clinical factors, such as edema, anemia, electrolyte disturbances, Human Immunodeficiency Virus (HIV), other infections or inflammation in general. Furthermore, although previous studies have documented that thymus atrophy is reversible when malnutrition is treated [5, 6], little is known about what determines thymus growth with nutritional rehabilitation. The aim of this study was therefore to investigate clinical factors associated with thymus size in children admitted for in-hospital treatment of SAM, and predictors of growth in thymus size with nutritional rehabilitation.

## Methods

### Study design

This study was an observational study among children admitted for in-hospital treatment of SAM between October 2012 and January 2013. The study was nested within the FeedSAM study, investigating physiological changes in children hospitalized with SAM, primarily change in plasma phosphate (P-phosphate). The FeedSAM study was registered in the ISRCTN registry with the number ISRCTN55092738.

### Study site and standard treatment

Mwanamugimu Nutrition Unit at Mulago Hospital is the main treatment center for children with complicated SAM in Uganda. At the time of the study, all children received in-patient treatment based on the Ugandan National Protocol for the Integrated Management of Acute Malnutrition, using World Health Organization (WHO)-recommended milk-based diets, F-75 and F-100 (Nutriset, France), as well as empiric parenteral antibiotics, usually ampicillin and gentamycin [9]. Dehydration was treated with oral rehydration solution for malnourished children (ReSoMal, Nutriset, France). When children were clinically well they were discharged to outpatient treatment with ready-to-use therapeutic food [10]. All biological mothers were offered routine counseling and testing for HIV antibodies, and if the mother was positive or absent, the child was tested. Antibody-positive children aged <18 months were referred for with PCR-based testing, according to WHO guidelines [11]. Diarrhea is a major concern at the unit, and at the time of the study, F-75 or F-100 were occasionally replaced with unfortified rice porridge for some days, when children had or developed diarrhea, and intolerance to the milk-based feeds was suspected.

### Inclusion and exclusion criteria

Inclusion criteria of children were: age 6–59 months; admission on weekdays for treatment of SAM, defined as either weight-for-length z-score (WLZ) < −3, using WHO Growth Standard [12], or mid-upper arm circumference (MUAC) <11.5 cm, or bilateral pitting edema; living close to the hospital; and a guardian providing informed consent. Exclusion criteria were: significant disability; manifest shock or severe respiratory distress requiring resuscitation at admission; hemoglobin <4 g/dl or a body weight <4.5 kg. Severe infections such as sepsis, HIV or tuberculosis were not reasons for exclusion. Inclusion and follow-up measurements were only possible when MJHR was present to perform the ultrasound measurements.

### Data collection

At admission, we obtained information about the child’s current symptoms and history using a structured questionnaire. Caretakers were asked to rate the perceived severity of their child’s illness on a visual analogue scale (VAS) from 1 to 10. Vital signs were noted (axillary temperature, pulse, respiratory rate, and capillary refill time), as well as edema and oral thrush. To assess appetite, we noted whether the child was able to consume all of the first served therapeutic feed.

Body weight was measured daily on a digital scale, to the nearest 100 g. Length and MUAC were measured to the nearest one mm, using an infant length board, and measurement tape, respectively. For analysis, anthropometric z-scores were computed using WHO Growth Standards [12]. In order to obtain the “true” body weight, after loss of edema, we used the lowest weight recorded after admission. We took into account that length was measured in all children by subtracting 0,7 cm from length measurements in children older than 2 years.

Study staff monitored children daily (on weekdays), recording weight, frequency and consistence of stools, type and amount of feed given, whether ReSoMal was given, and whether a naso-gastric tube was used for feeding. Children were classified as having diarrhea when passing three or more loose or watery stools per day.

### Thymus area measurement

**Fig. 1:**
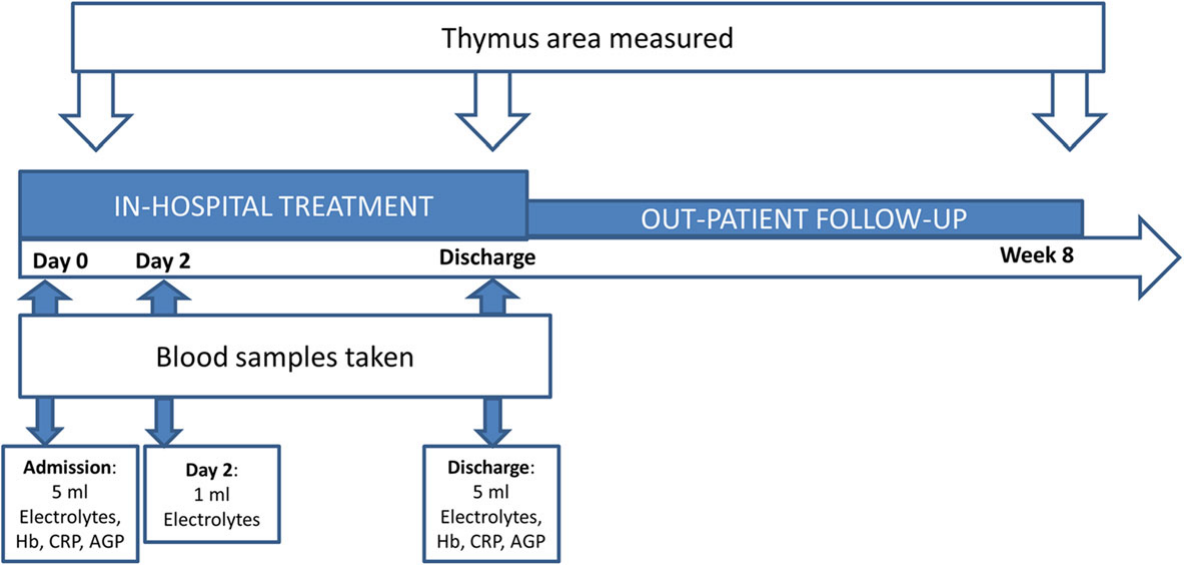
Overview of assessments in study (Hb: Haemoglobin; CRP: C-reactive protein; AGP: Alpha-1 acid glycoprotein)

**Fig. 2:**
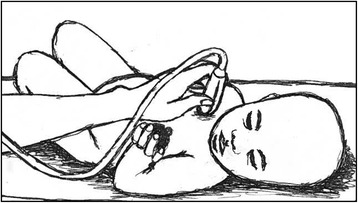
Measuring thymus size using ultrasound in a child

**Fig. 3:**
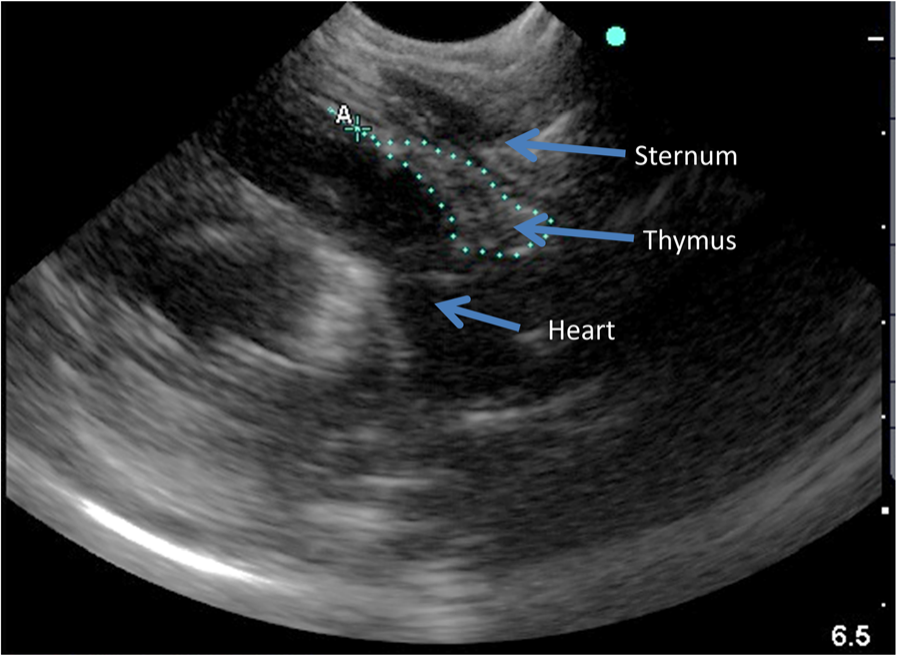
Ultrasound image of thymus in a malnourished child in the sagittal view. The line on the image traces the outline of the thymus, to measure the area

The same investigator (MJHR) measured thymus area at three time-points in all the children: One of the first days after admission (usually day 1 or 2), a few days before discharge from hospital, and at follow-up, approximately 8 weeks after admission (Fig. 1). MJHR had trained with a pediatric cardiologist experienced in thymus ultrasound (DLJ) at Copenhagen University Hospital Hvidovre in Denmark. DLJ supervised the data collection by reviewing selected ultrasound images send by email. Thymus size was measured using a portable ultrasound device (MicroMax, SonoSite, USA) with a pediatric abdominal probe. The child was lying on the back, in a bed or on the mothers lap, and the transducer was placed on the child’s chest, over the sternal bone, in a sagittal projection through the chest (Fig. 2). The thymus was identified as an echo-poor homogenous structure in the mediastinum, anterior to, and around the great vessels and the heart (Fig. 3). The largest lobe of the thymus was identified, and the area measured. Up to three area measurements were obtained per investigation, and the average calculated. In some cases, it was not possible to visualize the thymus.

### Blood sampling

Blood samples relevant to this study were collected from a peripheral vein at three time points: Sample one at admission before starting refeeding; sample two approximately 48 h after starting refeeding; and sample three, at the day of discharge to outpatient treatment. On all three occasions, 1 ml was collected in heparinized evacuated tubes, and on admission and discharge, 5 ml was also collected in a Cell Preparation Vacutainer® with citrate (Becton Dickinson, USA).

At admission and discharge, hemoglobin level was measured in heparinized full blood, using HemoCue® (Hb 201+, Ängelholm, Sweden). Citrate plasma was frozen at −80 C^o^, and shipped to Denmark on dry ice, where C-reactive protein (CRP) and α_1_-acid-glycoprotein (AGP), were measured at University of Copenhagen, Department of Nutrition, Exercise and Sports, using ABX Pentra® 400 (HORIBA, France). Heparinized plasma from all three time points was frozen to −20 C^o^ for up to 2 months, and inorganic phosphate (P-phosphate) was measured at Ebenezer Ltd Clinical Laboratory in Kampala (ISO 15189, Laboratory No. M0221), using molybdate UV method (Cobas Integra® 400 Plus).

### Control group

Apparently healthy children, with WLZ > −1 and aged 6–59 months, were recruited among children of hospital staff and siblings of hospitalized children and examined once. Thymus area assessment, physical examination, anthropometric measurements and blood sampling was done in controls, as described for the study patients.

### Statistics

Data were entered into EpiData (Odense, Denmark) and analyzed using Stata version 12 (StataCorp LP, College station, Texas, USA). Normally distributed variables were expressed as means ± standard deviations (SD), and variables that did not follow a normal distribution were expressed at medians and interquartile ranges (IQR). Two-sample t-tests were used to evaluate differences in means except in case of non-normally distributed outcomes where Mann-Whitney rank-sum- tests were used. Chi-square tests were used to compare proportions, except when the expected numbers were less than five, in which case Fisher’s exact test was used.

To identify correlates of thymus area on admission, while including children with an invisibly small thymus, we used an analysis of covariance (ANCOVA) allowing for left-censored measurements from children with an invisibly small thymus. Since area measurements <1 cm^2^ may be less accurate, we assigned all children with an undetectable thymus and children with measured area <1 cm^2^ to an unknown low value <1 cm^2^, and such left—censored measurements received less weight in the analysis as compared to accurately observed measurements, using the command “tobit” in Stata. Thymus area did not follow a normal distribution, and hence was logarithm-transformed (base 10); estimates were subsequently back-transformed. The analysis was adjusted for age and sex.

ANCOVA was also used to evaluate predictors of growth in thymus area at discharge and follow-up, defined as the change in thymus area (Δ thymus area = thymus area at follow-up – thymus area at admission). These analyses were adjusted for age, sex, number of days since first scan and thymus area on admission. Children with an undetectable thymus on admission were assigned a thymus area of 1 cm^2^ in order to calculate the change in thymus area over time.

As sensitivity analyses, we firstly analyzed correlates of thymus area and of predictors of growth in thymus area while only including children with a visible thymus, and without left-censoring of low values. Secondly, to assess if the associations were explained by body size, we analyzed correlates of thymus size while adjusting for body weight after loss of edema.

## Results

**Fig. 4:**
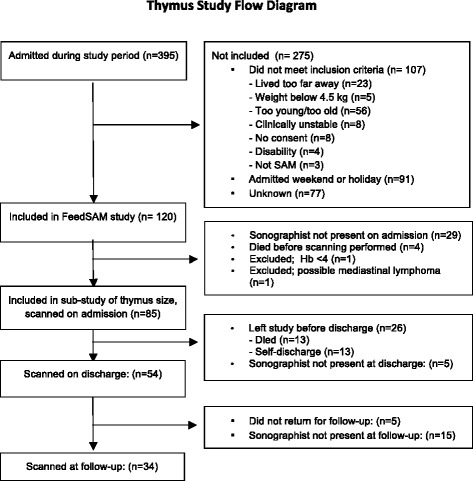
Flow diagram showing patients included and assessed at each time point

Of 120 children included in the FeedSAM study, 85 (71.7%) were included in the sub-study of thymus size measurement (Fig. 4). Of the children not included in this study, 29 were admitted on days on which the person performing the ultrasound scans was not present at the unit, 4 children died before ultrasound scans were performed, one was excluded before scanning due to a hemoglobin level <4 g/dl, and another child was excluded due to a suspected mediastinal lymphoma. The included 85 children had a median age of 16 months (IQR: 13; 23 months), 27 (32%) were girls, and 53 (62%) presented with edematous malnutrition. HIV status was unknown in 8 children (9%), and among the remaining, 15 (19%) were found to be HIV infected. Other findings from the full cohort of children have been described elsewhere [13–19].

In 22 (26%) of 85 children scanned on admission, the thymus was not visible by ultrasound. Children with an undetectable thymus had been rated more sick by their caretakers, were less likely to complete their first therapeutic feed (53 vs. 80%, *p* = 0.02), and had higher AGP (2.73 vs. 2.24 g/L, *p* = 0.02) (Table 1). P-phosphate was lower two days after admission in children with an undetectable thymus (1.30 vs. 1.55 mmol/L, *p* = 0.01). Respiratory symptoms (high respiratory rate or cough) did not differ in children with and without a detectable thymus.

**Table 1:** Anthropometric, clinical and biochemical characteristics of 85 children admitted with severe acute malnutrition by visibility of the thymus^a^

	n^b^	Thymus visible	Thymus not visible	P
		*n* = 63	*n* = 22	
Female sex	85	37 (23)	18 (4)	0.11
Age, months	85	16.0 (12.8; 22.7)	17.3 (13.2; 22.7)	0.59
Edema	85	62 (39)	64 (14)	0.88
Still breastfeeding	80	17(10)	14(3)	1.00
Anthropometric data				
Mid-upper arm circumference, cm	84	11.7 ± 1.4	11.6 ± 1.2	0.84
Weight-for-length z-score^c^	85	−3.4 ± 1.5	−3.5 ± 1.3	0.75
Weight-for-age Z-score^c^	85	−3.9 ± 1.2	−3.9 ± 1.1	0.92
Length-for-age Z-score	85	−3.1 ± 1.4	−3.0 ± 1.4	0.85
Clinical data				
HIV positive	77	16 (9)	32(6)	0.18
Symptoms reported by caretaker				
Diarrhea	81	42 (25)	45 (10)	0.80
Vomit	81	39 (23)	55 (12)	0.21
Cough	81	54 (32)	68 (15)	0.75
Fever	81	27 (22)	37 (6)	0.40
How sick according to caretaker^d^	80	6.4 ± 1.7	7.5 ± 2.2	0.03
Physical examination				
Pulse, B/min	81	138 ± 22	138 ± 21	0.95
Respiratory rate	80	38 ± 11	37 ± 10	0.67
Capillary refill time, sec	82	1.9 ± 0.8	2.1 ± 1.1	0.33
Temperature >37.5 °C	83	24 (15)	25 (5)	0.94
Oral thrush	71	26 (13)	29 (6)	0.82
Able to complete first feed	73	80 (43)	53 (10)	0.02
Biochemical data^e^				
Hemoglobin, g/dL	80	9.1 ± 2.3	8.7 ± 2.3	0.42
C-reactive protein, mg/L	65	19.4 (7.9; 37.3)	19.6 (12.8; 23.8)	0.19
> 5 mg/L	65	83 (40)	94 (16)	0.43
α_1_-acid glycoprotein, g/L	65	2.24 ± 0.72	2.73 ± 0.66	0.02
Sodium, mmol/L	81	138 ± 4	140 ± 5	0.03
Potassium, mmol/L	81	4.2 ± 0.7	4.0 ± 0.9	0.47
Inorganic phosphate, mmol/L				
Admission	82	1.07 ± 0.31	1.03 ± 0.29	0.63
Day two	72	1.55 ± 0.37	1.30 ± 0.35	0.01
Change during first two days	70	0.50 ± 0.38	0.31 ± 0.31	0.07

^a^Values presented are % (N), median (25%; 75%), or mean ± SD; Differences are considered significant when *p* < 0.05

^b^Number of children with factors recorded

^c^Using lowest weight recorded during admission, after loss of edema

^d^reported on a Visual Analogue Scale from 0 = perfectly healthy, to 10 = as sick as imaginable

^e^all values except hemoglobin measured in plasma

Of the 85 children scanned at admission, 54 were scanned at discharge, meaning that 31 were lost before discharge: 13 children died, 13 self-discharge before recommended, and five were discharged when the person performing the scans was not present at the unit. Before follow-up, another 20 children were lost from the study; 5 because they did not show up at follow-up, and 15 because the examiner was not present on the day of follow-up, leaving 34 with complete data at all three time points of the study (Fig. 4).

A greater proportion of those lost to follow-up were boys, while they did not differ significantly in terms of anthropometry, age, HIV-infection or acute-phase reactants. More of those lost to follow-up had an undetectable thymus on admission (35% vs. 12%, *p* = 0.02), but among those where it could be measured, thymus area was not different from those remaining in the study.

### Correlates of thymus size on admission

Among malnourished children with a visible thymus, thymus area ranged from 0.6 to 5.4 cm^2^, with a median of 1.3 cm^2^ on admission. In control children, thymus area ranged from 2.4 to 5.3 cm^2^, with a median of 3.5 cm^2^ (*p* < 0.001).

Thymus area correlated positively with most anthropometric indicators, including body weight, MUAC, weight-for-age z-score, WLZ, and length-for-age (Table 2). Caretaker-perceived severity of illness measured on the VAS scale correlated negatively with thymus area, and so did AGP (10^β^ = 0.73, 95% confidence interval (CI): 0.60; 0.90), meaning that thymus area was 27% lower per each g/l increase in AGP. Children with CRP > 5 mg/l had a 39% smaller thymus area than those with lower CRP (CI: −59%; −11%). The thymus was 27% smaller in HIV infected children, approaching significance (*p* = 0.06). Thymus area correlated with hemoglobin, and marginally with P-phosphate on the day of admission. On day two after admission, the correlation between P-phosphate and thymus area was stronger, with a 66% bigger thymus area per mmol/l higher P-phosphate (CI: 24%; 220%).

**Table 2:** Linear regression identifying factors associated with thymus area^a^ on admission among 85 children admitted with severe acute malnutrition. Invisible thymuses and values <1 cm^2^ censored as “below detection limit”^b^

	n^c^	10^β^ (95% confidence interval)	p
Female sex	85	1.18 (0.90; 1.54)	0.22
Age, months	85	0.99 (0.98; 1.01)	0.51
Edema present	85	1.22 (0.93; 1.58)	0.15
Still breastfeeding	80	1.09 (0.93; 1.58)	0.64
Anthropometric data			
Mid-upper arm circumference, cm	84	1.10 (1.01;1.19)	0.02
Weight-for-length, z-score^d^	85	1.11 (1.01; 1.21)	0.03
Weight-for-age, z-score^d^	85	1.13 (1.02; 1.25)	0.02
Length-for-age, z-score	85	1.07 (0.97; 1.19)	0.18
Clinical data, admission			
HIV positive	77	0.73 (0.53; 1.01)	0.06
Symptoms reported by caretaker			
Diarrhea	81	0.94 (0.71;1.22)	0.62
Vomit	81	0.86 (0.65; 1.13)	0.27
Cough	81	0.86 (0.66; 1.12)	0.27
Fever	81	0.97 (0.73; 1.29)	0.83
How sick according to caretaker^e^	80	0.89 (0.83; 0.95)	0.001
Physical examination on admission			
Pulse, beats/minute	81	1.00 (0.99; 1.00)	0.10
Respiratory rate, breaths/minute	80	1.00 (0.99; 1.00)	0.59
Capillary refill time, seconds	82	0.97 (0.84; 1.12)	0.68
Temperature >37.5°	83	0.80 (0.60; 1.08)	0.14
Oral thrush present	71	0.88 (0.98; 1.69)	0.43
Able to complete first feed	73	1.19 (0.86; 1.66)	0.29
Blood chemistry on admission^f^			
Hemoglobin, g/dL	80	1.08 (1.02; 1.14)	0.01
C-reactive protein > 5 mg/L	65	0.61 (0.41; 0.89)	0.01
α_1_-acid glycoprotein, g/L	65	0.73 (0.60; 0.90)	0.003
Sodium, mmol/L	81	0.99 (0.96; 1.02)	0.49
Potassium, mmol/L	81	1.01 (0.85; 1.21)	0.89
Inorganic phosphate, mmol/L			
Admission	81	1.50 (0.99; 2.27)	0.05
Day two	72	1.66 (1.24; 2.20)	0.001

^a^Thymus size is log_10_ of thymus area

^b^Data are back-transformed regression coefficients, adjusted for age and sex. Interpretation of e.g. 10 ^β^ = 1.08 is that by each unit increase in exposure variable, thymus area increases by 8%; Associations are considered significant when *p* < 0.05

^c^Number of children with factors recorded

^d^Using lowest weight recorded during admission, to account for loss of oedema

^e^Evaluated on a visual analogue scale from 1 to 10

^f^All values except hemoglobin measured in plasma

In the first sensitivity analysis, only including children with a visible thymus, we found overall similar associations. In the second sensitivity analysis, adjusting for body weight, age and sex, the associations were also similar, except there was no longer any significant association with WLZ or weight-for-age z-score (not shown in tables).

### Increase in thymus area

Children were admitted for a median of 16 days. Median thymus area increased from 1.3 to 1.6 cm^2^ (*p* = 0.006) at discharge, and to 2.5 cm^2^ (*p* < 0.001) at follow-up (Table 3). These figures were virtually unchanged when restricting the analysis to only children who were followed throughout the study. Despite the growth, thymus area at follow-up was still significantly smaller than thymus area in healthy children (*p* < 0.001). While the thymus was undetectable in 22 (26%) children on admission, this was the case for seven (13%) of 53 children at discharge, but in no children on follow-up, or in healthy controls.

**Table 3:** Thymus size and other characteristics in children during treatment of severe acute malnutrition, and in a group of healthy children^a^

	Malnourished children during treatment			Healthy children
	Admission	Discharge	Follow-up	
No. of children scanned	85	54	34	20
No. of children with visible thymus	74 (63)	87 (47)	100 (34)	100 (20)
Thymus area, cm^2 b^	1.3 (1.0; 1.7)	1.6 (1.4; 2.1)	2.5 (2.1; 3.3)	3.5 (3.1; 3.8)
Time from admission scanned, days	1 (1; 1)	14 (12; 20)	56 (50; 57)	-
Time admitted, days	-	16 (13;22)		-
Weight gain, g/kg/day	-	5.9 (3.7; 8.3)	4.7 (3.0; 6.4)	-
Weight, kg	6.8 ± 1.5	7.6 ± 1.4	8.5 ± 1.3	11.2
Length, cm	72.7 ± 5.7	72.2 ± 5.2	73.6 ± 5.6	80.7 ± 9.3
Weight-for-age, z-score^c^	- 3.9 ± 1.2	- 3.1 ± 1.1	−2.2 ± 1.0	0.0 ± 1.0
Length-for-age, z-score	- 3.1 ± 1.4	−3.4 ± 1.2	−3.2 ± 1.1	−0.9 ± 1.2
Weight-for-length, z-score^c^	- 3.4 ± 1.4	- 1.78 ± 1.1	−0.7 ± 0.9	0.6 ± 0.9
< −2 and > −3	23 (20)	22 (12)	9 (3)	0 (0)
< − 3	60 (52)	17 (9)	0 (0)	0 (0)
Mid-upper arm circumference, cm	11.6 ± 1.4	12.0 ± 1.1	12.9 ± 1.1	14.9
Boys	66 (69)	65 (35)	56 (19)	55 (11)
Age, months	16.0 (13.0; 22.7)	16.7 (13.5; 23.9)	17.8 (14.9; 26.0)	20.6 (12.0; 34.4)
Currently breastfeeding	16 (13)	-	-	50 (10)
Hemoglobin, g/dl	9.0 ± 2.3	9.7 ± 1.9	-	10.2 ± 1.5
Plasma C-reactive protein, mg/L	19.6 (8.8; 31.2)	0.4 (0.2; 1.9)	-	0.8 (0.2;2.8)
Plasma α_1_-acid glycoprotein, g/L	2.37 ± 0.73	1.09 ± 0.39	-	0.83 ± 0.30

^a^Values presented are n, %(n), median (25%; 75%) or mean ± SD

^b^Only including children with a visible thymus

^c^Admission z-scores were computed for all children based on the lowest weight recorded (after loss of edema)

Few factors were associated with increase in thymus area during nutritional rehabilitation (Table 4). Hemoglobin level measured on admission was positively associated with increase in thymus area at discharge, and AGP was negatively associated with growth in thymus area at follow-up. The only anthropometric indicator associated with thymus growth was increase in MUAC, with 0.20 cm^2^ higher increase in thymus area per cm higher increase in MUAC at discharge (CI: 0.05 cm^2^; 0.36 cm^2^), and at follow-up (0.29 cm^2^ higher increase in thymus size per cm higher increase in MUAC, CI: 0.04 cm^2^; 0.54 cm^2^). Children given unfortified rice porridge in hospital had a 0.67 cm^2^ smaller increase in thymus area at follow-up (CI: −1.28 cm^2^; −0.05 cm^2^). The sensitivity analyses showed similar associations, only including children with a visible thymus on admission.

**Table 4:** Correlates of change in thymus size from admission to discharge and to follow-up among children treated for severe acute malnutrition^a^

	To discharge			To follow-up		
	n^b^	β (95%CI)	p	n^b^	β (95% CI)	p
Female sex	47	0.00 (−0.30;0.31)	0.98	34	−0.06 (−0.65; 0.53)	0.84
Age, months	47	0.01 (−0.01; 0.03)	0.40	34	- 0.02 (−0.06; 0.01)	0.19
Days from admission	47	0.02 (−0.01; 0.04)	0.21	34	0.00 (−0.04; 0.05)	0.94
Clinical data, admission						
Edema present	47	−0.14 (−0.50;0.23)	0.46	34	−0.34 (−0.97; 0.29)	0.28
HIV infected	46	0.09 (−0.38; 0.55)	0.71	33	0.35 (−0.60; 1.31)	0.45
Still breastfeeding	43	0.08 (−0.38;0.54)	0.71	31	0.25 (−0.66; 1.17)	0.58
How sick according to caretaker^c^	46	0.04 (−0.05; 0.13)	0.35	34	0.07 (−0.13;0.28)	0.46
Physical examination, admission						
Temperature >37.5°	46	0.01 (−0.43; 0.45)	0.96	34	0.10 (−0.66; 0.86)	0.79
Capillary refill time, sec	45	−0.05 (−0.24; 0.14)	0.62	32	0.14 (−0.34; 0.62)	0.55
Able to complete first feed	44	−0.04 (−0.40; 0.32)	0.81	31	0.35 (−0.45; 1.15))	0.37
Blood chemistry, admission						
C-reactive protein >5 mg/L	38	0.36 (−0.07; 0.79)	0.10	26	−0.22 (−1.16; 0.72)	0.63
α_1_-acid glycoprotein, g/L	38	0.03 (−0.24; 0.31)	0.81	26	−0.60 (−1.12; −0.08)	0.03
Hemoglobin, g/dL	46	0.08 (0.01; 0.15)	0.02	33	−0.00 (−0.14; 0.13)	0.95
Inorganic phosphate, mmol/L	46	0.16 (−0.34; 0.66)	0.52	33	0.12 (−1.01; 1.24)	0.83
Anthropometric growth in same period						
Weight gain rate, kg/day^d^	47	5.77 (−1.20; 12.74)	0.10	34	14.02 (−8.12; 36.15)	0.21
Δ mid-upper arm circumference, cm	46	0.20 (0.05;0.36)	0.01	23	0.29 (0.04; 0.54)	0.03
Δ weight-for-length z-score^d^	47	−0.02 (−0.20; 0.17)	0.87	34	0.08 (−0.19; 0.34)	0.57
Δ weight-for-age z-score^d^	47	0.08 (−0.25; 0.41)	0.62	34	0.22 (−0.20; 0.63)	0.30
Observations and treatments given during admission						
Diarrhea observed	47	- 0.10 (−0.44; 0.25)	0.58	34	0.10 (−0.52; 0.71)	0.75
Rice porridge given	46	−0.11 (−0.42; 0.19)	0.47	33	−0.67 (−1.28; −0.05)	0.03
Naso-gastric tube used	47	−0.08 (−0.44; 0.28)	0.67	34	−0.25 (−0.92; 0.42)	0.46

^a^Data shown are regression coefficients of linear regression analysis of change in thymus size (Δ thymus size) adjusted for thymus size on admission, days since admission, age and sex. Children with invisible thymus on admission were assumed to have thymus area = 1 cm^2^; Interpretation of e.g. β = 0.20 means a 0.20 cm^2^ further increase in thymus size per unit increase in exposure variable; Associations are considered significant when *p* < 0.05

^b^n = number of children in whom data is available

^c^Evaluated on a visual analogue scale from 1 to 10

^d^Weight gain = present weight – lowest weight during admission, to account for loos of oedema

## Discussion

Thymus atrophy has previously been reported among malnourished children, based on autopsy studies [4] and in ultrasound studies [5, 6]. It has been hypothesized that thymus atrophy could reflect the immune deficiency of malnutrition, causing greater susceptibility to infections in malnourished children [3]. In community studies of children from Guinea Bissau and Bangladesh [7, 8] children with a small thymus had a higher mortality risk, indicating that thymus size could be a marker of immune competence, or perhaps just a marker of good health or robustness. However, factors determining thymus size in children with SAM, or thymus growth with nutritional rehabilitation, have not previously been reported.

Using thymus area as a marker, we found that thymus size was positively associated with most anthropometric indicators of nutritional status. Similar observations have been done among apparently healthy children in Guinea Bissau [7], Gambia [20] and Bangladesh [21], and they confirm the concept of the thymus being a “barometer of malnutrition” [22]. We found that thymus size was negatively associated with acute phase reactants in plasma. This could both reflect acute infections and chronic, low-grade inflammation. Previous studies have suggested that acute infections such as malaria [7], and neonatal infections [23] can cause thymus atrophy, and inflammation has recently been reported as a major risk factor for death in children with severe malnutrition [24]. The fact that thymus size was reduced in children with CRP above just 5 mg/l could suggest that low-grade chronic inflammation could also reduce thymus size, although distinguishing between infections and inflammation may be somewhat speculative. Our study confirms that thymus size is reduced by nutritional insults and infections. Both cause elevated levels of cortisol, which animal studies have found to cause thymus atrophy [25], similar to low levels of leptin [26]. We saw a negative association with care-giver reported severity of illness, similar to a study among newborn children in Guinea Bissau indicating that children who were “not well” according to their mothers, had a smaller thymus, and suggested thymus size to be a general “barometer of good health” [27]. Although malnutrition has been associated with other immune abnormalities, like altered lymphocyte numbers and function, in children [28], as well as in animal experiments [29], it is still not known whether thymus size is actually linked to immune function, or whether the association between thymus size and risk of dying is caused by other non-immunological confounding factors, such as diagnosed or undiagnosed infections.

As children admitted with SAM are often both infected and malnourished, it is not surprising that 27% of children in our study had an undetectable thymus on admission. Similarly, an autopsy study of severely malnourished children reported how their thymus was reduced to “an irregular strand of fibrous tissue” [4]. Ultrasound invisibility of the thymus could also be caused by hyper-inflated lungs, (caused by e.g. pneumonia), preventing ultrasound penetration. However, neither reported cough, nor respiratory rate was significantly different in children with or without a visible thymus, suggesting that pneumonia may not be an important cause in this cohort. It is possible that the thymus in some cases would have been visualized in the hands of a more experienced sonographer. However, the fact that we saw similar associations when including invisible thymuses as “invisibly small”, and when including only visible thymuses (in the sensitivity analysis) suggests that it may be reasonable to assume that thymuses were undetectable because they were very small.

Thymus size was positively associated with hemoglobin level, and P-phosphate, which to our knowledge, has not previously been reported. The greater association with phosphate on day two may be explained by the fact that most ultrasound scans were done one or two days after admission, and therefore closer in time to the second blood sample. Infections may cause both anemia and hypophosphatemia [30], and thus the association could reflect the effect of inflammation on thymus size. However, the associations persisted after adjusting for CRP and AGP. Anemia and hypophosphatemia may be markers of poor nutritional status, although their associations with thymus size remained in the sensitivity analysis adjusting for body size. It is also plausible that hypophosphatemia by itself may contribute to thymus atrophy. Hypophosphatemia has been found to cause leukocyte dysfunction [31], and phosphorus is a type II nutrient, essential for growth and maintenance of lean body mass [32]. Thymus atrophy occurs in animals deficient in other type II nutrients, like zinc [33] and magnesium [34], and a recent study found thymus size in low-birth-weight infants to be associated with zinc levels in cord-blood [35]. However, the association between phosphorous status and immune function has until now not received much attention.

The thymus was smaller in HIV-infected children, although not statistically significant (*p* = 0.06). It has previously been reported that predominantly well-nourished children with HIV have a smaller thymus [36]. Our findings could be due to low power, with only 15 HIV-positive children in the study.

We found no association between nutritional edema and thymus size. Thymus atrophy is known to occur in children with kwashiorkor [4], and one study from Egypt reported that children with edematous malnutrition had a smaller thymus that those with non-edematous malnutrition [6]. Others have found higher CD4 counts in edematous than non-edematous children with malnutrition [37], suggesting that these children had better immunity; however, our data does not indicate that this is reflected by a larger thymus.

Breastfed children did not have a larger thymus, in contrast to previous studies [38]. This could be due to low power, since only 13 of the 85 children were breastfed, or because the association between breastfeeding and thymus size seems to be greatest around 4 months of age, when breastfeeding is exclusive.

Although thymus size recovered with nutritional rehabilitation, it was still significantly smaller than in well-nourished children. This could be because the children’s nutritional status was still poorer that the healthy controls, or because immunological recovery may take longer than anthropometric recovery, as previously suggested [39].

Few factors predicted change in thymus area. The only anthropometric indicator, in which growth was associated with thymus growth, was MUAC. MUAC reflects muscle mass, and this suggests that recovery of thymus size correlates with recovery of muscle mass. Similar observations were done in a study from Bolivia, where zinc supplementation accelerated both growth in MUAC and growth in thymus size [40].

Interestingly, children in whom F75 had been replaced by unfortified rice porridge at some point during in-patient treatment had reduced thymus growth at follow-up. Rice porridge was given to about half of the children, during their stay in hospital, when children had diarrhea that worsened after starting refeeding. Confounding by indication is an obvious possibility, but most other indicators of disease severity, such as observed diarrhea, reported severity of illness or capillary refill time were not associated with reduced thymus growth. Rice porridge contains very little vitamins and minerals, virtually no fat, and most of its energy is derived from carbohydrates. In this same cohort, we previously reported that giving rice porridge was associated with lower increase in phosphate over the first two days of nutritional rehabilitation [14], and also with higher risk of death in hospital [19]. Considering the association between P-phosphate and thymus size, it seems plausible that that the reduced growth could be a consequence of replacing the therapeutic diets with rice porridge. Rice porridge is not part of any established protocol for treatment of SAM or diarrhea, and the practice was stopped at Mwanamugimu Nutrition Unit shortly after the study.

There are a number of limitations to this study: First, its observational design means that we cannot draw firm conclusions about causality. The exploratory approach and the use of multiple testing also means that our findings may only be used to generate hypotheses which should be confirmed in other studies. Second, the most commonly method used previously to measure thymus size is the *thymic index*, obtained by multiplying the diameter by the area [41]. However, likely due to the severe thymic atrophy, we were mostly unable to visualize the thymus in the transversal projection or to measure the diameter. Therefore, we had to rely on the area measurement only. Our results can therefore not be compared directly to studies using thymic index. Interestingly, other studies of thymus size in children with SAM have, like us, used the thymus area [5], and our experience suggest that this is the method of choice in children with SAM. Third, we had a rather large loss-to-follow-up, giving low power in the follow-up analyses, and limiting the generalizability of this data, since the children remaining in the study are likely to differ from those lost to follow-up.

## Conclusion

A significant proportion of children hospitalized with SAM have no detectable thymus by ultrasound, and these children appear to be sicker than those with a visible thymus. Thymus size is positively associated with anthropometric indicators of nutritional status, with hemoglobin level and phosphate in plasma, and negatively associated with acute phase reactants in plasma. Growth in thymus size at follow-up seems to be associated with increase in MUAC. Children whose F-75 is partially replaced by unfortified cereal may have poorer recovery of thymus size. Our results emphasize the importance of phosphorous in the diets for malnourished children, and suggests further research into the role of phosphate for thymus size and function in children [42].

## Abbreviations

AGP: α-1 acid glycoprotein
ANCOVA: Analysis of co-variance
CI: 95% Confidence Interval
CRP: C-reactive protein
HIV: Human Immunodeficiency Virus
IQR: Inter-quartile range
MUAC: Mid-upper-arm circumference
P-: Plasma-
SAM: Severe acute malnutrition
WHO: World Health Organization
WLZ: Weight-for-length z-score
